# Association between 15 known or potential breast cancer susceptibility genes and breast cancer risks in Chinese women

**DOI:** 10.20892/j.issn.2095-3941.2021.0358

**Published:** 2021-10-12

**Authors:** Fenfen Fu, Dongjie Zhang, Li Hu, Senthil Sundaram, Dingge Ying, Ying Zhang, Shuna Fu, Juan Zhang, Lu Yao, Ye Xu, Yuntao Xie

**Affiliations:** 1 Department of Breast Surgery, Peking University International Hospital, Beijing 102206, China; 2 Key Laboratory of Carcinogenesis and Translational Research (Ministry of Education), Familial & Hereditary Cancer Center, Peking University Cancer Hospital & Institute, Beijing 100142, China; 3 Prenetics Limited, Hong Kong 999077, China; 4 Beijing CircleDNA Gene Technology Co., Ltd., Beijing 100020, China

**Keywords:** Multigene panel sequencing, susceptibility genes, breast cancer risk, phenotype, case-control study

## Abstract

**Objective:**

There are many hereditary breast cancer patients in China, and multigene panel testing has been a new paradigm of genetic testing for these patients and their relatives. However, the magnitude of breast cancer risks related to multiple breast cancer susceptibility genes are largely unknown in Chinese women.

**Methods:**

We screened pathogenic variants in 15 established or potential breast cancer susceptibility genes from 8,067 consecutive Chinese female breast cancer patients and 13,129 Chinese cancer-free female controls. These breast cancer patients were unselected for age at diagnosis or family history.

**Results:**

We found that pathogenic variants in *TP53* [odds ratio (OR): 16.9, 95% confidence interval (CI): 5.2–55.2]; *BRCA2* (OR: 10.4, 95% CI: 7.6–14.2); *BRCA1* (OR: 9.7, 95% CI: 6.3–14.8); and *PALB2* (OR: 5.2, 95% CI: 3.0–8.8) were associated with a high risk of breast cancer. *ATM*, *BARD1, CHEK2*, and *RAD51D* were associated with a moderate risk of breast cancer with ORs ranging from 2-fold to 4-fold. In contrast, pathogenic variants of *NBN*, *RAD50, BRIP1*, and *RAD51C* were not associated with increased risk of breast cancer in Chinese women. The pathogenic variants of *PTEN, CDH1*, and *STK11* were very rare, so they had a limited contribution to Chinese breast cancer. Patients with pathogenic variants of *TP53, BRCA1, BRCA2*, and *PALB2* more often had early-onset breast cancer, bilateral breast cancer, and a family history of breast cancer and/or any cancer.

**Conclusions:**

This study provided breast cancer risk assessment data for multiple genes in Chinese women, which is useful for genetic testing and clinical management of Chinese hereditary breast cancer.

## Introduction

Breast cancer is the most frequent malignant tumor in Chinese women, with approximately 268,600 newly-diagnosed cases per year. Furthermore, the burden of breast cancer is still increasing^1–3^. It is estimated that nearly 10% of unselected breast cancer patients in China carry pathogenic variants in cancer susceptibility genes^4^. Detecting these pathogenic variants and precisely estimating their risks for breast cancer will provide the basis for prevention and management of hereditary breast cancers. Advances in sequencing technology have made multigene testing, or “panel testing,” a routine option to detect pathogenic variants for potential breast cancer patients and their relatives. These panels usually contain established breast cancer susceptibility genes, such as *BRCA1, BRCA2, PALB2, TP53, CHEK2*, and *ATM*, and potential breast cancer susceptibility genes, such as *BARD1, BRIP1, RAD50, RAD51C*, and *RAD51D*^5,6^. However, the magnitude of breast cancer risks associated with these known or potential breast cancer genes is largely unknown in Chinese women.

Large case-control association studies that quantify the breast cancer risks of multiple genes from panel testing have been mainly conducted in Caucasian women or other populations^7–14^. However, these data may not be generally applicable to Chinese women. Although the frequencies of pathogenic variants of breast cancer genes in Chinese women are comparable to those in other ethnicities^4,15,16^, studies by our group or other groups have shown substantial differences in the spectrum of pathogenic variants of breast cancer susceptibility genes between Chinese and non-Chinese ethnicities, with up to one-third of the pathogenic variants in Chinese women not being found in Caucasian women^17–22^. Moreover, early-onset breast cancer (i.e., diagnosed at or before the age of 40 years) is more common in Chinese women than Caucasian women^1^. Therefore, the breast cancer risks in specific genes in Chinese women may differ from those in other populations. In this study, we therefore screened pathogenic variants in 15 established or potential breast cancer susceptibility genes in 8,067 unselected Chinese female breast cancer patients and 13,129 Chinese cancer-free female controls, then compared the risk-related phenotypes between the patients with pathogenic variants and those without a pathogenic variant. We aimed to determine the breast cancer risks of the 15 breast cancer genes in Chinese women.

## Materials and methods

### Study population

A total of 8,085 consecutive breast cancer patients who were treated at the Breast Center of Peking University Cancer Hospital & Institute from October 2003 to May 2015 underwent 62-gene panel sequencing, as described in our previous report^4^. Eighteen male breast cancer patients were excluded from the analysis, and the remaining 8,067 female breast cancer patients were included in this study. Early-onset breast cancer patients were defined as patients diagnosed at or before the age of 40 years. Family history of breast cancer was defined as the breast cancer patient having 1 or more breast cancer patients in the first-, second-, or third-degree relatives, and family history of any cancer was defined as the breast cancer patient having 1 or more cancer patients (any kind of cancer) in the first-, second-, or third-degree relatives. The family history of breast or other cancer was collected from medical records and/or telephone interviews. A total of 13,129 Chinese women (ages ≥ 18 years) without a personal history of any cancer were recruited from the general population and were considered as a reference control for this case-control study. Written informed consents were obtained from all participants. This study was reviewed and approved by the Ethics Committee of Peking University Cancer Hospital & Institute (Approval No.2011041205) and was performed in accordance with the Declaration of Helsinki.

### Sequencing assay

For the breast cancer cohort, genomic DNA extracted from peripheral blood was used for 62-gene panel sequencing at an average depth of 200-fold coverage of the target region, as described in our previous study^4^. We selected 15 established or potential breast cancer susceptibility genes from the 62-gene panel for further analysis based on published studies^5,7,23,24^, including 10 established breast cancer susceptibility genes (*BRCA1, BRCA2, PALB2, TP53, PTEN, CDH1, STK11, ATM, CHEK2*, and *NBN*) and 5 potential breast cancer susceptibility genes (*BARD1, BRIP1, RAD50, RAD51C*, and *RAD51D*). For the control cohort, genomic DNA extracted from buccal swabs was used for whole-exome sequencing at an average depth of 100-fold coverage of the target region. Reads were aligned to the reference human genome, GRCh37. Germline variations were called with GATK. Annotations were defined using ANNOVAR (https://annovar.openbioinformatics.org/en/latest/).

### Variant classification

In this study, we analyzed germline variants in the 15 established or potential breast cancer susceptibility genes. Only variants with < 1% population frequency in the population databases including 1,000 Genomes (https://www.1000genomes.org), NHLBI Exome Sequencing Project (ESP6500, https://evs.gs.washington.edu/EVS/), and the Exome Aggregation Consortium (ExAC, https://exac.broadinstitute.org) were collected. Among these, the truncating variants (nonsense and frameshift variants) were included in this study, but the truncating variants in the last 55 base pairs of the penultimate exon or last exon that potentially avoided nonsense-mediated messenger RNA decay and did not influence known functional domains, were excluded. For splice-site, synonymous, nonsynonymous, in-frame, and stop-loss variants, only variants classified as pathogenic or likely pathogenic by ClinVar (http://www.ncbi.nlm.nih.gov/clinvar/) were included in the analysis. The variants with conflicting interpretations of pathogenicity in ClinVar were further annotated according to the ACMG/AMP standards and guidelines^25^, with supporting data from function prediction software, public literature, and curated databases (**Supplementary Table S1**). Variants classified to be pathogenic or likely pathogenic were considered as pathogenic in this study.

### Statistical analysis

Case-control association analysis within each gene was performed using logistic regression. The strength of associations with breast cancer was estimated by the odds ratio (OR) and corresponding 95% confidence interval (CI). Genes were categorized as high risk (OR ≥ 5.0), moderate risk (2.0 ≤ OR < 5.0), or of no clinical relevance (OR < 2.0)^7^. Categorical variables between mutation carriers and noncarriers were compared using the chi-square test or Fisher’s exact test, where appropriate. Continuous variables were tested using a *t*-test. Two-sided *P* values less than 0.05 were considered to be statistically significant. All analyses were performed using SPSS 20.0 statistical software for Windows (SPSS, Chicago, IL, USA).

## Results

### Characteristics of the study population

The 8,067 consecutive Chinese female breast cancer patients analyzed in this study were unselected for age at diagnosis or family history. The median age at diagnosis for breast cancer patients was 50 years (range: 19–98 years) (**Table 1**). Among them, 18.2% were diagnosed at or before the age of 40 years (early-onset breast cancer), and 10.0% had a family history of breast cancer. A total of 13,129 Chinese women without a personal history of cancer were enrolled as controls. The median age at entry was 33 years (range: 18–84 years) (**Table 1**). The majority of breast cancer cases (95.8%) and controls (93.6%) were of Chinese Han descent (**Table 1**).

**Table 1:** Characteristic of the study population

	Breast cancer cases	Cancer-free controls
No. of individuals	8,067	13,129
Age (median and range)	50 (19–98)	33 (18–84)
Age (mean ± SD)	51.1 ± 11.6	34.8 ± 9.3
Ethnicity		
Han	7,727 (95.8%)	12,288 (93.6%)
Non-han	340 (4.2%)	841 (6.4%)

### Pathogenic germline variants detected in breast cancer cases and controls

Pathogenic germline variants of the 15 genes were found in 654 (8.11%) breast cancer cases and 251 (1.91%) controls (**Table 2**). Fourteen breast cancer patients and 1 control had pathogenic variants in 2 different genes (double variant carriers; data not shown). For breast cancer cases, *BRCA2* (284, 3.52%) was the most frequently mutated gene, followed by other established breast cancer susceptibility genes: *BRCA1* (146, 1.81%), *PALB2* (57, 0.71%), and *TP53* (31, 0.38%). For controls, *BRCA2* (46, 0.35%) still ranked as the most frequently mutated gene, followed by the candidate genes, *RAD50* (31, 0.24%) and *BRIP1* (29, 0.22%). Notably, pathogenic germline variants of *PTEN*, *CDH1*, and *STK11* were extremely rare in Chinese breast cancer patients or controls (**Table 2**). Furthermore, in all 533 pathogenic germline variants of the 15 genes analyzed in this study, 385 (72.2%) variants have been reported in the latest version of the ClinVar dataset (**Supplementary Table S2**).

**Table 2:** Breast cancer risks of the 15 genes estimated by case-control association analysis in Chinese women

Gene	Case (*n* = 8,067)		Control (*n* = 13,129)		OR (95% CI)	*P*
	No. of carriers	%	No. of carriers	%		
*TP53*	31	0.38%	3	0.02%	16.9 (5.2–55.2)	1.72 × 10^−10^
*BRCA2*	284	3.52%	46	0.35%	10.4 (7.6–14.2)	3.13 × 10^−73^
*BRCA1*	146	1.81%	25	0.19%	9.7 (6.3–14.8)	1.71 × 10^−37^
*PALB2*	57	0.71%	18	0.14%	5.2 (3.0–8.8)	1.21 × 10^−11^
*BARD1*	15	0.19%	8	0.06%	3.1 (1.3–7.2)	7.27 × 10^−3^
*CHEK2*	26	0.32%	17	0.13%	2.5 (1.4–4.6)	2.45 × 10^−3^
*RAD51D*	31	0.38%	23	0.18%	2.2 (1.3–3.8)	3.37 × 10^−3^
*ATM*	31	0.38%	24	0.18%	2.1 (1.2–3.6)	5.12 × 10^−3^
*PTEN*	5	0.06%	0	0.00%	–	–
*CDH1*	1	0.01%	0	0.00%	–	–
*STK11*	1	0.01%	1	0.01%	1.6 (0.1–26.0)	1.00
*NBN*	6	0.07%	5	0.04%	2.0 (0.6–6.4)	0.35
*RAD50*	21	0.26%	31	0.24%	1.1 (0.6–1.9)	0.73
*BRIP1*	11	0.14%	29	0.22%	0.6 (0.3–1.2)	0.17
*RAD51C*	2	0.02%	22	0.17%	0.1 (0.0–0.6)	2.69 × 10^−3^
In total	654^#^	8.11%	251^#^	1.91%	–	–

^#^Fourteen breast cancer patients and 1 cancer-free control carrying pathogenic variants in 2 different genes. OR, odds ratio; CI, confidence interval. OR and *P* values were estimated using logistic regression.

### Risk estimation in the 15 genes based on this case-control study

Breast cancer risks for each of the genes were estimated by comparing the frequency of pathogenic variants identified in breast cancer cases to controls. Logistic regression results showed that 8 genes in this study were significantly associated with increased risk of breast cancer in Chinese women (**Table 2**). Among these genes, *TP53* (OR: 16.9, 95% CI: 5.2–55.2); *BRCA2* (OR: 10.4, 95% CI: 7.6–14.2); *BRCA1* (OR: 9.7, 95% CI: 6.3–14.8); and *PALB2* (OR: 5.2, 95% CI: 3.0–8.8) were classified as high risk breast cancer susceptibility genes in Chinese women (**Table 2**). *BARD1* (OR: 3.1, 95% CI: 1.3–7.2); *CHEK2* (OR: 2.5, 95% CI:1.4–4.6); *RAD51D* (OR: 2.2, 95% CI: 1.3–3.8); and *ATM* (OR: 2.1, 95% CI: 1.2–3.6) were classified as moderate risk breast cancer risk genes (**Table 2**). The 8 high or moderate risk susceptibility genes accounted for 7.5% of unselected Chinese breast cancer patients in this study (5.2% for *BRCA1/2* and 2.3% for the other genes). In contrast, *NBN*, *RAD50, BRIP1*, and *RAD51C* were not associated with increased risks of breast cancer in Chinese women (**Table 2**). In addition, pathogenic variants of *PTEN* (5 cases), *CDH1* (1 case), and *STK11* (1 case and 1 control) were too rare to estimate their risks of breast cancer in Chinese women (**Table 2**). The breast cancer risks of susceptibility genes were higher after being adjusted for age (**Supplementary Table S3**).

We further estimated breast cancer risks for each of the genes based on pathogenic truncating variants (**Supplementary Table S4**). Among these*, BRCA1, BRCA2*, and *PALB2* were still classified as high risk, and *ATM, BARD1*, *CHEK2*, and *RAD51D* were still classified as moderate risk breast cancer susceptibility genes in Chinese women (**Supplementary Table S4**). As the majority of pathogenic variants in the *TP53* gene were missense, the truncating variants in the *TP53* gene were too rare to estimate the risk of breast cancer in this study (**Supplementary Table S4**).

We further analyzed the breast cancer risks of the 15 genes stratified by estrogen receptor (ER), progesterone receptor (PR), and human epidermal growth factor 2 status in this case-control study. Among the ER and/or PR^+^ and HER2^-^ subgroups, *TP53* (OR: 15.9, 95% CI: 4.6–54.6); *BRCA2* (OR: 12.8, 95% CI: 8.9–17.7); *BRCA1* (OR: 5.6, 95% CI: 3.5–9.2); and *PALB2* (OR: 6.0, 95% CI: 3.4–10.5) were still classified as high risk breast cancer susceptibility genes (**Table 3**). *CHEK2* (OR: 2.5, 95% CI: 1.2–5.0) and *ATM* (OR: 2.7, 95% CI: 1.5–4.9) were classified as moderate risk susceptibility genes (**Table 3**), while *BARD1* and *RAD51D* were not significantly associated with risk in this subgroup (**Table 3**). Among the HER2^+^ group, only *TP53* (OR: 23.8, 95% CI: 6.5–86.6) was classified as a high risk breast cancer susceptibility gene, while *BRCA2* (OR: 4.5, 95% CI: 2.8–7.2); *BRCA1* (OR: 3.4, 95% CI: 1.7–6.8); *PALB2* (OR: 3.2, 95% CI: 1.4–7.3); and *CHEK2* (OR: 3.8, 95% CI: 1.7–8.5) were classified as moderate risk breast cancer susceptibility genes (**Table 3**). Among the triple negative subgroup, *TP53, BRCA1, BRCA2, PALB2, BRAD1*, and *RAD51D* were all classified as high risk breast cancer susceptibility genes (**Table 3**). Of these, *BRCA1* had the highest risk of breast cancer susceptibility (OR: 42.1, 95% CI: 26.8–66.2) (**Table 3**).

**Table 3:** Breast cancer risks of the 15 genes stratified by ER, PR, and HER2 status of breast cancer cases

Gene	ER and/or PR+, and HER2-cases (*n* = 4,421)		HER2+ cases (*n* = 1,847)		ER-, PR-, and HER2-cases (*n* = 1,103)		ER/PR/HER2 unknown cases (*n* = 696)	
	No. of carriers (%)	OR (95% CI)	No. of carriers (%)	OR (95% CI)	No. of carriers (%)	OR (95% CI)	No. of carriers (%)	OR (95% CI)
*TP53*	16 (0.36)	15.9 (4.6–54.6)	10 (0.54)	23.8 (6.5–86.6)	2 (0.18)	7.9 (1.3–47.6)	3 (0.43)	18.9 (3.8–94.0)
*BRCA2*	190 (4.30)	12.8 (8.9–17.7)	29 (1.57)	4.5 (2.8–7.2)	44 (3.99)	11.8 (7.8–17.9)	21 (3.02)	8.8 (5.3–14.9)
*BRCA1*	47 (1.06)	5.6 (3.5–9.2)	12 (0.65)	3.4 (1.7–6.8)	82 (7.43)	42.1 (26.8–66.2)	5 (0.72)	3.8 (1.4–9.9)
*PALB2*	36 (0.81)	6.0 (3.4–10.5)	8 (0.43)	3.2 (1.4–7.3)	13 (1.18)	8.7 (4.2–17.8)	0 (0.00)	–
*BARD1*	4 (0.09)	1.5 (0.4–4.9)	2 (0.11)	1.8 (0.4–8.4)	8 (0.73)	12.0 (4.5–32.0)	1 (0.14)	2.3 (0.3–18.9)
*CHEK2*	14 (0.32)	2.5 (1.2–5.0)	9 (0.49)	3.8 (1.7–8.5)	1 (0.09)	0.7 (0.1–5.3)	2 (0.29)	2.2 (0.5–9.6)
*RAD51D*	11 (0.25)	1.4 (0.7–2.9)	6 (0.32)	1.9 (0.8–4.6)	12 (1.09)	6.3 (3.1–12.6)	2 (0.29)	1.6 (0.4–7.0)
*ATM*	22 (0.50)	2.7 (1.5–4.9)	5 (0.27)	1.5 (0.6–3.9)	1 (0.09)	0.5 (0.1–3.7)	3 (0.43)	2.4 (0.7–7.9)
*PTEN*	4 (0.09)	–	1 (0.05)	–	0 (0.00)	–	0 (0.00)	–
*CDH1*	0 (0.00)	–	0 (0.00)	–	1 (0.09)	–	0 (0.00)	–
*STK11*	1 (0.02)	3.0 (0.2–47.5)	0 (0.00)	–	0 (0.00)	–	0 (0.00)	–
*NBN*	5 (0.11)	3.0 (0.9–10.3)	1 (0.05)	1.4 (0.2–12.2)	0 (0.00)	–	0 (0.00)	–
*RAD50*	14 (0.32)	1.3 (0.7–2.5)	3 (0.16)	0.7 (0.2–2.3)	3 (0.27)	1.2 (0.4–3.8)	1 (0.14)	0.6 (0.08–4.5)
*BRIP1*	6 (0.14)	0.6 (0.3–1.5)	3 (0.16)	0.7 (0.2–2.4)	2 (0.18)	0.8 (0.2–3.4)	0 (0.00)	–
*RAD51C*	0 (0.00)	–	2 (0.11)	0.6 (0.2–2.7)	0 (0.00)	–	0 (0.00)	–

ER, estrogen receptor; PR, progesterone receptor; HER2, human epidermal growth factor receptor 2; OR, odds ratio; CI, confidence interval.

### The impact of breast cancer susceptibility genes on clinical characteristics

Pathogenic variants in breast cancer susceptibility genes are usually associated with risk-related phenotypes, such as early-onset breast cancer, bilateral breast cancer, and higher frequencies of family cancer history^26^. We therefore compared these risk-related characteristics between the patients with pathogenic variants and those without any pathogenic variant. Double variant carriers were excluded from the clinical characteristic analysis.

The mean age at diagnosis of breast cancer patients with pathogenic variants of *TP53* (41.8 years *vs.* 51.4 years, *P* = 5.00 × 10^−6^); *BRCA2* (47.8 years *vs.* 51.4 years, *P* = 2.95 × 10^−7^); *BRCA1* (44.7 years *vs.* 51.4 years, *P* = 1.01 × 10^−11^); *PALB2* (47.6 years *vs.* 51.4 years, *P* = 0.02); and *RAD51D* (46.3 years *vs.* 51.4 years, *P* = 0.02) was significantly younger than that of noncarriers (**Table 4**). Similarly, pathogenic variants of *TP53, BRCA2, BRCA1*, and *RAD51D* were significantly associated with early-onset breast cancer (**Table 4**), and pathogenic variants of *TP53, BRCA2, BRCA1*, and *NBN* were significantly associated with premenopausal breast cancer (**Table 4**). Among these, the *TP53* gene, which conferred the highest risk of breast cancer, was also associated with the youngest mean age at diagnosis and the highest frequency of early-onset breast cancer (**Table 4**). In addition, the high risk genes (*TP53, BRCA2*, and *BRCA1)* were significantly associated with bilateral breast cancer (**Table 4**). When comparing the family history of cancer, all high risk and moderate risk genes except for *RAD51D* were significantly associated with higher frequencies of family history of breast cancer and/or family history of any cancer (**Table 4**). In summary, all 8 high or moderate risk susceptibility genes in this study affected 1 or more risk-related phenotypes. In contrast, the 4 genes that were not associated with increased risk of breast cancer in this study (*NBN, RAD50, BRIP1*, and *RAD51C*) had no effect on any of the risk-related phenotypes (**Table 4**).

**Table 4:** Comparison of risk-related clinical characteristics between patients with germline pathogenic variant and noncarriers

Gene	All cases	Age at diagnosis		Early-onset breast cancer (≤ 40 years)		Premenopausal breast cancer		Bilateral breast cancer		Positive family history of breast cancer		Positive family history of any cancer	
		Mean ± SD	*P*	No. of patients (%)	*P*	No. of patients (%)	*P*	No. of patients (%)	*P*	No. of patients (%)	*P*	No. of patients (%)	*P*
Non-carriers	7,413	51.4 ± 11.6	Ref	1,276 (17.2)	Ref	3,839 (51.8)	Ref	184 (2.5)	Ref	626 (8.4)	Ref	2,323 (31.3)	Ref
*TP5*3	30	41.8 ± 12.1	5.00 × 10^−6^	13 (43.3)	1.61 × 10^−4^	26 (86.7)	1.36 × 10^−4^	5 (16.7)	8.51 × 10^−4^	4 (13.3)	0.32	16 (53.3)	9.60 × 10^−3^
*BRCA*2	279	47.8 ± 10.8	2.95 × 10^−7^	75 (26.9)	3.10 × 10^−5^	163 (58.4)	0.03	30 (10.8)	1.64 × 10^−16^	85 (30.5)	1.13 × 10^−35^	143 (51.3)	2.60 × 10^−12^
*BRCA*1	141	44.7 ± 9.3	1.01 × 10^−11^	50 (35.5)	1.68 × 10^−8^	108 (76.6)	5.15 × 10^−9^	10 (7.1)	1.58 × 10^−3^	55 (39.0)	3.84 × 10^−36^	89 (63.1)	1.06 × 10^−15^
*PALB2*	52	47.6 ± 11.4	0.02	13 (25.0)	0.14	32 (61.5)	0.16	1 (1.9)	1.00	9 (17.3)	0.04	31 (59.6)	1.20 × 10^−5^
*BARD1*	14	50.4 ± 9.3	0.73	2 (14.3)	1.00	6 (42.9)	0.50	0 (0.0)	1.00	2 (14.3)	0.76	9 (64.3)	0.02
*CHEK2*	24	50.3 ± 8.4	0.64	3 (12.5)	0.73	16 (66.7)	0.15	1 (4.2)	0.45	4 (16.7)	0.28	13 (54.2)	0.03
*RAD51D*	28	46.3 ± 11.7	0.02	10 (35.7)	0.02	19 (67.9)	0.09	0 (0.0)	1.00	1 (3.6)	0.56	12 (42.9)	0.19
*AT*M	29	49.8 ± 13.6	0.45	8 (27.6)	0.14	18 (62.1)	0.27	2 (6.9)	0.16	6 (20.7)	0.04	18 (62.1)	3.75 × 10^−4^
*PTEN*	5	47.6 ± 7.0	0.46	0 (0.0)	0.60	3 (60.0)	1.00	2 (40.0)	0.01	1 (20.0)	0.36	1 (20.0)	1.00
*CDH1*	1	46.0	0.64	0 (0.0)	1.00	1 (100.0)	1.00	0 (0.0)	1.00	0 (0.0)	1.00	0 (0.0)	1.00
*STK11*	1	46.0	0.64	0 (0.0)	1.00	1 (100.0)	1.00	0 (0.0)	1.00	0 (0.0)	1.00	0 (0.0)	1.00
*NBN*	6	43.8 ± 4.3	0.11	1 (16.7)	1.00	6 (100.0)	0.03	0 (0.0)	1.00	0 (0.0)	1.00	2 (33.3)	1.00
*RAD50*	19	48.6 ± 14.2	0.29	6 (31.6)	0.18	10 (52.6)	1.00	0 (0.0)	1.00	2 (10.5)	1.00	4 (21.1)	0.33
*BRIP1*	9	45.9 ± 9.3	0.15	4 (44.4)	0.09	7 (77.8)	0.18	1 (11.1)	0.20	1 (11.1)	0.55	3 (33.3)	1.00
*RAD51C*	2	54.0 ± 8.5	0.75	0 (0.0)	1.00	0 (0.0)	0.23	0 (0.0)	1.00	0 (0.0)	1.00	1 (50.0)	0.53
In Total	8,053^#^	51.1 ± 11.6	–	1,461 (18.1)	–	4,263 (52.9)	–	236 (2.9)	–	796 (9.9)	–	2,665 (33.1)	–

^#^Fourteen patients carrying pathogenic variants in 2 different genes were excluded from clinical characteristics analysis. SD, standard deviation; BBC, bilateral breast cancer.

*TP53, PTEN*, *STK11*, and *CDH1* are associated with Li-Fraumeni syndrome, Cowden syndrome, Peutz-Jeghers syndrome, and hereditary diffuse gastric cancer syndrome^27–30^. Among the 31 breast cancer patients with the *TP53* pathogenic variant in this study, only 3 patients (9.7%) met the criteria for the Li-Fraumeni syndrome. After excluding the 3 patients with Li-Fraumeni syndrome, the *TP53* gene was still significantly associated with a high risk of breast cancer in Chinese women (OR: 15.2, 95% CI: 4.6–50.1, *P* = 2.00 × 10^−9^), which suggested that women with *TP53* pathogenic variants but without a family history of Li-Fraumeni syndrome still had a high risk of breast cancer. None of the 5 breast cancer patients with the *PTEN* variant met the Cowden syndrome criteria, and the patient with *STK11* variant did not meet the Peutz-Jeghers criteria. The patient with the *CDH1* variant in this study did not present with lobular breast cancer, nor with a personal history or family history of gastric cancer, although there were 255 (3.2%) invasive lobular breast cancer patients in this study.

## Discussion

In this study, we screened pathogenic variants in 15 established or potential breast cancer susceptibility genes in 8,067 Chinese unselected female breast cancer patients and 13,129 Chinese cancer-free female controls. Our results classified *TP53, BRCA1, BRCA2*, and *PALB2* as high risk, and *ATM, BARD1*, *CHEK2*, and *RAD51D* as moderate risk breast cancer susceptibility genes in Chinese women. In contrast, our study revealed that *NBN*, *RAD50, BRIP1*, and *RAD51C* were not associated with an increased risk of breast cancer, and *PTEN, CDH1*, and *STK11* had a very limited contribution to Chinese breast cancer.

We found that the *TP53* gene conferred the highest risk of breast cancer in Chinese women (OR: 16.9), and patients carrying pathogenic variants of the *TP53* gene had the youngest mean age at diagnosis. Pathogenic variants of the *TP53* gene were also associated with a high risk of breast cancer in Japanese women^9^. However, 2 recent large-scale case-control studies of Caucasian women reported that the *TP53* gene was not significantly associated with breast cancer risk^13,14^. This difference might be explained by the observation that the prevalence of *TP53* pathogenic variants in Asian breast cancer patients was much higher than that in Caucasian breast cancer patients^9,13,14,31^. In addition, a previous study based on patients with Li-Fraumeni syndrome reported a very high relative risk of breast cancer (OR: 105) for the *TP53* gene^27^. This difference might be explained by that the *TP53* variant carriers in our study were identified from unselected breast cancer patients, and only a minority of them met the Li-Fraumeni Chompret criteria. Importantly, our results showed that Chinese women with *TP53* pathogenic variants but without a family history of Li-Fraumeni syndrome had a high risk of breast cancer (OR = 15.2).

This case-control study showed approximately a 10-fold increased risk of breast cancer for *BRCA1/2* pathogenic variant carriers, when compared with noncarriers in Chinese women. Recent case-control studies in Caucasian women have reported relative risks of breast cancer for the *BRCA1* and *BRCA2* genes of 5.9–10.6-fold and 3.3–5.9-fold, respectively^8,13,14^, which were lower than the magnitude of breast cancer risks estimated in this study. This difference might be explained by sample selection and ethnic differences.

The *PALB2* gene was recently classified as a high risk breast cancer susceptibility gene in Caucasian women, with 53% cumulative risk to age 80 years and 7-fold relative risk for female breast cancer^7,32^, which was comparable to the breast cancer risk of the *BRCA2* gene in Caucasian women^8,13,33^. However, our results and a recent study from another group^34^ both suggested that *PALB2* conferred 5-fold increased risk for breast cancer in Chinese women, indicating that the risk associated with the *PALB2* gene was lower than that of *BRCA1/2* genes in Chinese women, although the magnitude of risk for the *PALB2* gene reached the threshold of high risk genes.

This study classified *ATM, BARD1*, *CHEK2*, and *RAD51D* as moderate risk breast cancer susceptibility genes in Chinese women. Although *ATM* and *CHEK2* were identified as classic moderate risk genes in Caucasian women^35^, the risks of the 2 genes in Chinese women are still worth investigating because it has been reported that the risk of pathogenic variants of the 2 genes was highly related to location and the type of variants, and considerable differences in the spectrum of *ATM* and *CHEK2* variants were observed between Chinese and Caucasian ethnicities. For example, the dominant negative *ATM* p.Val2424Gly variant confers a much higher risk than truncating variants of the *ATM* gene (OR: 8.0–11.0 for p.Val2424Gly and OR: 2.2 for truncating variants)^35–37^. *CHEK2* c.1100delC, the most frequent truncating variant in Caucasian women, confers a higher risk than the common missense mutation, *CHEK2* p.Ile157Thr^38–40^. However, these specific variants, such as *ATM* p.Val2424Gly and *CHEK2* c.1100delC, were absent in Chinese women^20,21^. In summary, our study defined *ATM* and *CHEK2* as moderate risk genes for breast cancer in Chinese women. Some large case-control studies in Caucasian women showed that *BRAD1* and *RAD51D* were breast cancer susceptibility genes^7,13^, while other studies did not reach this conclusion^8,14^. Our results indicated that *BRAD1* and *RAD51D* were moderate risk breast cancer susceptibility genes in Chinese women. In addition, breast cancer patients with the *BRAD1* and *RAD51D* pathogenic variants were associated with a higher frequency of family cancer history and early-onset breast cancer, respectively. These clinical characteristics also suggested that *BARD1* and *RAD51D* were breast cancer susceptibility genes in Chinese women.

The pathogenic variants of *PTEN, CDH1*, and *STK11* were too rare in our study to estimate their risks of breast cancer in Chinese women, which suggested that these genes had a very limited contribution to breast cancer in unselected Chinese women. In addition, the pathogenic variant carriers for *PTEN, CDH1*, and *STK11* identified from unselected breast cancer patients in this study did not meet the criteria for the Cowden syndrome, hereditary diffuse gastric cancer syndrome, and Peutz-Jeghers syndrome, respectively. Additional larger case-control studies or segregation analysis in families might be needed to fully elucidate the breast cancer risks for *PTEN, CDH1*, and *STK11* in Chinese women*. NBN* serves as an established breast cancer susceptibility gene in the latest guidelines, because many studies reported that *NBN* c.657del5 (a founder variant in the Slavic population) was associated with increased risk of breast cancer^41,42^. However, *NBN* c.657del5 was absent in Chinese breast cancer patients and cancer-free controls in this study, and our results showed that other truncating variants of *NBN* were not associated with breast cancer risk. Our study did not support *RAD50, BRIP1* and *RAD51C* as breast cancer susceptibility genes in the Chinese population, consistent with results from case-control studies in Caucasian women^7,8,11,14^.

This study also showed that breast cancer susceptibility genes conferred different risks in breast cancer molecular subgroups. For example, pathogenic variants in *BRCA1* conferred the highest breast cancer risk in the triple negative subgroup than that in other subgroups. Pathogenic variants in *TP53* conferred the highest breast cancer risk in the HER2^+^ subgroup than that in other subgroups. In addition, the *ATM* and *CHEK2* genes were classified as moderate risk susceptibility genes in the ER and/or PR^+^ and HER2^-^ subgroups, but were not associated with a risk in the triple negative subgroup. These findings were consistent with previous case-control studies^8,12–14^.

This study had some limitations. The ages of the cancer-free controls were younger than those of the breast cancer cases. It therefore may have underestimated the risks based on logistic regression models. However, our previous study based on the kin-cohort method found that the cumulative breast cancer risks in *BRCA1/2* carriers up to the age of 70 years were 37.9% and 36.5% in Chinese women, respectively^43^, which were also 10-fold higher risks than noncarriers (3.6% by the age of 70 years). Similar results based on 2 different methods confirmed the reliability of our findings, although some bias existed in this case-control study.

## Conclusions

This large case-control study classified *TP53, BRCA2, BRCA1*, and *PALB2* as high risk, and *ATM, BARD1*, *CHEK2*, and *RAD51D* as moderate risk breast cancer susceptibility genes in Chinese women. In contrast, the study showed that *NBN*, *RAD50, BRIP1*, and *RAD51C* were not associated with an increased risk of breast cancer, and *PTEN, CDH1*, and *STK11* had a very limited contribution to Chinese breast cancer. These results suggested that the 8 high or moderate risk susceptibility genes should be covered in panel testing for high risk Chinese breast cancer patients and their relatives. Overall, our risk assessment data should be useful for the prevention and early detection of Chinese women who carry a pathogenic variant in the 8 breast cancer susceptibility genes.
