# Supplementary material for: Association between 15 known or potential breast cancer susceptibility genes and breast cancer risks in Chinese women
Source: Cancer Biol Med. 2021 Oct 12;19(2):253–62. doi: 10.20892/j.issn.2095-3941.2021.0358 (PMC8832954; doi:10.20892/j.issn.2095-3941.2021.0358)
Supplement: Supplementary file 1 [file cbm-19-253-s001.pdf]

Supplementary materials

Table S1 The variants with conflicting interpretations of pathogenicity in ClinVar were further annotated according to the ACMG/AMP standards and guidelines

AAChange.ref Gene	ClinVar submission	ClinVar class	BRCA exchange class	BIC class	HGMD class	HGMD phenotype	Publications	ACMG evidence summary	ACMG conclusion
ATM: NM_000051.3: c.6154G>A: p.Glu2052Lys	Pathogenic(2), Likely pathogenic(3), Uncertain significance(2)	Conflicting Interpretations Of pathogenicity	–	–	DM	Breast and/or ovarian cancer	PMID: 23946315, PMID: 27616075, PMID: 28492532, PMID: 10330348, PMID: 25572163, PMID: 25741868, PMID: 20301790	PP5_strong, PM2	Likely pathogenic
BRCA2: NM_000059.3: c.8350C>T: p.Arg2784Trp	Likely pathogenic(4), Uncertain significance(4)	Conflicting Interpretations Of pathogenicity	Not Yet Reviewed Clinically Importance	Clinically Importance: unknown	DM?	Breast and/or ovarian cancer	PMID: 10923033; PMID19043619; PMID: 33471991; PMID: 27616075; PMID: 19200354; PMID: 18451181	PM1+PM2+PS_ PP5_M	Likely pathogenic
RAD51D: NM_002878.3: c.620C>T: p.Ser207Leu	Likely pathogenic(7), Uncertain significance(3)	Conflicting Interpretations Of pathogenicity	–	–	DM	Ovarian cancer	PMID: 22986143; PMID: 26845104; PMID: 33471991	PM2+PP3+PS_PP5_M	Likely pathogenic
BRCA1: NM_007294.3: c.442-22_442-13del	Pathogenic(1), Likely pathogenic(1), Uncertain significance(1)	Conflicting Interpretations Of pathogenicity	Not Yet Reviewed Clinically Importance	No record	No record	No record	PMID: 33471991; PMID: 31214711	PM2+PS_PP5_M	Likely pathogenic
RAD51C: NM_058216.3: c.934C>T: p.Arg312Trp	Likely pathogenic(2), Uncertain significance(3)	Conflicting Interpretations Of pathogenicity	–	–	DM	Ovarian cancer	PMID: 22810696; PMID: 28492532; PMID: 28829762; PMID: 25741868	PM2,PP5_ moderate,PP3	Likely pathogenic
BRIP1: NM_032043.2: c.751C>T: p.Arg251Cys	Likely pathogenic(2), Uncertain significance(3)	Conflicting Interpretations Of pathogenicity	–	–	DM	Fanconi anaemia/ Breast cancer	PMID: 23613520; PMID: 31214711; PMID: 33471991	PM1+PM2+PP3+PS_ PP5_M	Likely pathogenic
TP53: NM_000546.5: c.845G>A: p.Arg282Gln	Likely pathogenic(2), Uncertain significance(5)	Conflicting Interpretations Of pathogenicity	–	–	DM	Neuroblastoma	PMID: 10864200; PMID: 33471991	PM2+PP3+PS_PP5_M	Likely pathogenic

Table S1 Continued

AAChange.ref Gene	ClinVar submission	ClinVar class	BRCA exchange class	BIC class	HGMD class	HGMD phenotype	Publications	ACMG evidence summary	ACMG conclusion
TP53: NM_000546: c.C472T: p.R158C	Pathogenic(1), Likely pathogenic(3), Uncertain significance(5)	Conflicting Interpretations Of pathogenicity	–	–	DM	Adrenocortical carcinoma	PMID: 12826609; PMID: 29979965; PMID: 22170717; PMID: 29958926; PMID: 23200980	PS3+PM1+PS_PP5_M+BS1	Likely pathogenic
CHEK2: NM_007194.3: c.1283C>T: p.Ser428Phe	Pathogenic(10), Likely pathogenic(5), Uncertain significance(1)	Conflicting Interpretations Of pathogenicity	–	–	DM	Breast cancer	PMID: 25741868; PMID: 26845104; PMID: 22419737; PMID: 15649950; PMID: 16551709; PMID: 16914568; PMID: 18085035; PMID: 18571837; PMID: 27153395	PS3+PM2+PS_PP5	Pathogenic
CHEK2: NM_007194.3: c.917G>C: p.Gly306Ala	Likely pathogenic(4), Uncertain significance(2)	Conflicting Interpretations Of pathogenicity	–	–	DM	Breast cancer	PMID: 22419737; PMID: 33471991	PM1+PS_PP5	Likely pathogenic
CHEK2: NM_007194.3: c.470T>C: p.Ile157Thr	Pathogenic(9), Likely pathogenic(8), Uncertain significance(3)	Conflicting Interpretations Of pathogenicity	–	–	DM	Breast cancer	PMID: 25741868; PMID: 20729852; PMID: 21514219; PMID: 23946381; PMID: 24728327; PMID: 27424552; PMID: 24244489; PMID: 26510858; PMID: 27392074; PMID: 24599715; PMID: 27230571; PMID: 26264438; PMID: 27747004; PMID: 26845104 PMID: 27438779; PMID: 24880342; PMID: 27632928; PMID: 10617473; PMID: 12533788; PMID: 15239132; PMID: 15492928;	PS3+PM1+PS_PP5_M+BS1	Likely pathogenic

Table S1 Continued

AAChange.ref Gene	ClinVar submission	ClinVar class	BRCA exchange class	BIC class	HGMD class	HGMD phenotype	Publications	ACMG evidence summary	ACMG conclusion
							PMID: 16816021; PMID: 17085682; PMID: 17517688; PMID: 19442246; PMID: 21244692; PMID: 19492346; PMID: 27696107; PMID: 25798211; PMID: 27711073; PMID: 16671833; PMID: 21504591		

BIC, Breast Cancer Information Core; HGMD, Human Gene Mutation Database; DM, disease causing mutation; ACMG/AMP, The American College of Medical Genetics and Genomics and the Association for Molecular Pathology.

Table S2 The percentages of variants in this study reported in the ClinVar database

	No. of variants identified from cases and controls	No. of variants in ClinVar* (%)	No. of variants out ClinVar* (%)
BRCA2	197	148 (75.1%)	49 (24.9%)
BRCA1	97	81 (83.5%)	16 (16.5%)
PALB2	55	32 (58.2%)	23 (41.8%)
ATM	44	28 (63.3%)	16 (36.4%)
TP53	25	20 (80.0%)	5 (20.0%)
CHEK2	28	20 (71.4%)	8 (28.6%)
BARD1	14	9 (64.3%)	5 (35.7%)
RAD50	16	11 (68.8%)	5 (31.3%)
BRIP1	22	14 (63.6%)	8 (36.4%)
RAD51D	11	6 (54.5%)	5 (45.5%)
PTEN	4	3 (75.0%)	1 (25.0%)
NBN	9	4 (44.4%)	5 (55.6%)
RAD51C	8	7 (87.5%)	1 (12.5%)
CDH1	1	0 (0.0%)	1 (100.0%)
STK11	2	2 (100.0%)	0 (0.0%)
In total	533	385 (72.2%)	148 (27.8%)

*Compared with the ClinVar dataset, version 20210501.

Table S3 Breast cancer risks of susceptibility genes estimated by case-control association analysis and adjusted for age in Chinese women

Gene	Case (*n* = 8,067)		Control (*n* = 13,129)		Adjusted OR (95% CI)	Adjusted *P*-value
	No. of carriers	%	No. of carriers	%		
TP53	31	0.38%	3	0.02%	56.6 (16.1–198.4)	2.86×10^{-10}
BRCA1	146	1.81%	25	0.19%	14.6 (8.9–23.7)	6.32×10^{-27}
BRCA2	284	3.52%	46	0.35%	14.1 (9.7–20.4)	9.02×10^{-45}
PALB2	57	0.71%	18	0.14%	5.5 (3.0–10.0)	2.73×10^{-8}
ATM	31	0.38%	24	0.18%	4.3 (2.0–9.2)	1.76×10^{-4}
BARD1	15	0.19%	8	0.06%	3.5 (1.1–10.8)	0.03
CHEK2	26	0.32%	17	0.13%	3.2 (1.4–7.2)	5.07×10^{-3}
RAD51D	31	0.38%	23	0.18%	2.4 (1.2–4.7)	9.34×10^{-3}
PTEN	5	0.06%	0	0.00%	–	–
CDH1	1	0.01%	0	0.00%	–	–
STK11	1	0.01%	1	0.01%	1.3 (0.1–20.5)	0.86
NBN	6	0.07%	5	0.04%	2.5 (0.6–10.0)	0.19
RAD50	21	0.26%	31	0.24%	1.5 (0.7–2.9)	0.28
BRIP1	11	0.14%	29	0.22%	0.6 (0.3–1.2)	0.15
RAD51C	2	0.02%	22	0.17%	0.2 (0.0–0.8)	0.02
In total	654[#]	8.11%	251[#]	1.91%	–	–

[#]Fourteen breast cancer patients and 1 cancer-free control carrying pathogenic variants in 2 different genes. OR, odds ratio; CI, confidence interval. OR and *P* values were estimated by logistic regression.

Table S4 Estimated breast cancer risks of susceptibility genes based on pathogenic truncating variants

Gene	Case (n = 8,067)		Control (n = 13,129)		OR (95% CI)	P
	No. of carriers	%	No. of carriers	%		
TP53	4	0.05%	0	0.00%	–	–
BRCA1	263	3.26%	38	0.29%	11.6 (8.3–16.3)	1.77×10^{-70}
BRCA2	136	1.69%	18	0.14%	12.5 (7.6–20.4)	5.06×10^{-38}
PALB2	57	0.71%	16	0.12%	5.8 (3.3–10.2)	1.72×10^{-12}
ATM	15	0.19%	8	0.06%	3.1 (0.3–7.2)	0.007
BARD1	24	0.30%	9	0.07%	4.4 (2.0–9.4)	4.00×10^{-5}
CHEK2	31	0.38%	22	0.17%	2.3 (1.3–4.0)	0.002
RAD51D	28	0.35%	20	0.15%	2.3 (1.3–4.1)	0.004
PTEN	5	0.06%	0	0.00%	–	–
CDH1	1	0.01%	0	0.00%	–	–
STK11	1	0.01%	1	0.01%	1.6 (0.1–26.0)	1.00
NBN	6	0.07%	4	0.03%	2.4 (0.7–8.7)	0.27
RAD50	21	0.26%	30	0.23%	1.1 (0.7–2.0)	0.65
BRIP1	10	0.12%	27	0.21%	0.6 (0.3–1.2)	0.17
RAD51C	1	0.01%	16	0.12%	0.1 (0.01–0.8)	0.006
In total	593[#]	7.35%	208[#]	1.58%	–	–

[#]Ten breast cancer patients and 1 cancer-free control carrying pathogenic variants in 2 different genes. OR, odds ratio; CI, confidence interval. OR and *P* values were estimated using logistic regression.
